# Heterogeneity in topographic control on velocities of Western Himalayan glaciers

**DOI:** 10.1038/s41598-018-31310-y

**Published:** 2018-08-27

**Authors:** Lydia Sam, Anshuman Bhardwaj, Rajesh Kumar, Manfred F. Buchroithner, F. Javier Martín-Torres

**Affiliations:** 1 Institut für Kartographie, Technische Universität Dresden, Dresden, Germany; 2 Department of Environmental Science, Sharda University, Greater Noida, India; 3 Division of Space Technology, Department of Computer Science, Electrical and Space Engineering, Luleå University of Technology, Luleå, Sweden; 4 Instituto Andaluz de Ciencias de la Tierra (CSIC-UGR), Armilla, Granada Spain; 5 UK Centre for Astrobiology, School of Physics and Astronomy, University of Edinburgh, Edinburgh, UK

## Abstract

Studies of the seasonal and annual patterns of glacier velocities improve our understanding of the ice volume, topography, responses to climate change, and surge events of glaciers. Such studies are especially relevant and equally rare for the Himalayan glaciers, which supply many rivers that sustain some of the most heavily populated mountainous regions in the world. In particular, the control of the hypsometric distribution of geomorphometric parameters, such as slope, aspect, and curvature, on the dynamics of Himalayan glaciers have never been studied so far, at the river basin scale. Here, we present the degree to which topographic and hypsometric parameters affect the seasonal and annual average flow velocities of 112 glaciers in the Baspa River basin in the Western Indian Himalaya by analysing Global Land Ice Velocity Extraction from Landsat 8 (GoLIVE) datasets for the years 2013–2017. We observe, (i) significant heterogeneity in topographic controls on the velocities of these glaciers, (ii) elevation and the seasons play important roles in regulating the degree to which morphometric parameters (slope, aspect, and curvature) affect these velocities, (iii) a possible polythermal regime promoting both sliding and deformational forms of motion in a majority of these glaciers, and (iv) a detailed analysis of complex topographic controls within various elevation zones using a novel hypso-morphometric approach. These findings can help us to better model the dynamics of Himalayan glaciers and their responses to the future climatic scenarios. The inferences also suggest the need to incorporate dynamic topography in glacio-hydrological models in the wake of constant glacial evolutions.

## Introduction

Global glacier monitoring is pertinent for observing the direct impacts of changes in climate on water security and future sea levels^1^. Although mountain glaciers constitute only ~3% of the global glacial area^2^, the need for precise areal and volumetric estimations of mountain glaciers is well established; they contribute immensely to sea-level rise, owing to their latitudinal vulnerability and rapid melting rates under present climate scenarios^2–5^. A particular need to focus on the Hindu Kush-Himalayan (HKH) glaciers arises because these glaciers represent ~50% (by area)^6^ of all of the glaciers outside of the poles, and their meltwater sustains a downstream population of ~1.3 billion people^6,7^. The amplified occurrence of extreme weather events^8^ and glacial disasters^9–12^ in the HKH mountains further emphasise the need for extensive glacio-hydro-meteorological database generation and research in the coming years^13^. However, the uncertainties in the understanding of the status and future of glaciers and climate change in the Himalayan region are high^14^, owing to the scarcity and fragmentary nature of the applicable glacio-meteorological records^6,7,15^, a geopolitical reluctance to share data^16^, and the extreme terrain and inclement climate of the region, which complicates the efforts of glacio-hydrological measurements^17,18^. Assessments and predictions of the recession of the HKH glaciers and their hydrology using presently available datasets are alarming and indicate a disastrous future for the region^19^.

Seasonal and annual estimates of glacier velocities can be useful in assessing glacier ice thicknesses and bed topography^20^, mass changes^21^, glacier retreats and advances^22^, glacial erosion^23^, and glacier flow regimes^24^. Moreover, investigating the patterns and estimates of glacier flows with respect to their surface topography can further increase our understanding of the topographic controls on glacier motion^25^. Such attempts have been made to study surge-type glaciers in Svalbard^26^, the Scandinavian ice sheets^27^, past ice sheets^28^, and the Greenland ice sheet^29^. However, barring a few attempts that consider the slope and distribution of elevation^23,30^, such studies are lacking for the Himalayan glaciers. Particularly, a holistic study that takes into account all the primary morphometric and topographic parameters, such as slope, aspect, curvature, and distribution of elevation is completely missing in the Himalayan context. Most of the Himalayan glaciers, with a few exceptions^14^, are undergoing rapid recession and the related morphological changes^31,32^, leading to changes in their flow regimes^33^. Definitive information on topographic controls on glacier flows can be extremely informative in predicting the future dynamics of glaciers.

In the present study, we attempt to fill several research gaps in our understanding of topographic controls over Himalayan glacier velocities by unravelling the heterogeneity in the effects of various topographic parameters on glacier flow. (i) The analyses encompass all the primary morphometric and topographic parameters, such as slope, aspect, curvature, and elevation distribution. (ii) To derive reliable conclusions, we normalise the climatic and geological variability by analysing all of the glaciers within a single river basin; thus, only the effects of topography are emphasised. (iii) We perform the analyses on seasonal time scales (i.e., for the pre-melt, melting, and post-melt seasons) so that the variations in the velocities in areas with the same topography in different seasons can be observed. (iv) Using field observations, we provide suitable thresholds for the peak correlation values and the differences in correlation values between the primary and secondary peaks derived from the Global Land Ice Velocity Extraction from Landsat 8 (GoLIVE) data^34,35^ for the years 2013–2017 to obtain reliable, basin-scale velocities for such analyses for the first time for Himalayan glaciers. Finally, (v) we also suggest hypso-morphometric analyses as a novel approach where the control of morphometric parameters is separately evaluated for different hypsometric zones in order to better disentangle the contributions from topography.

**Figure 1:**
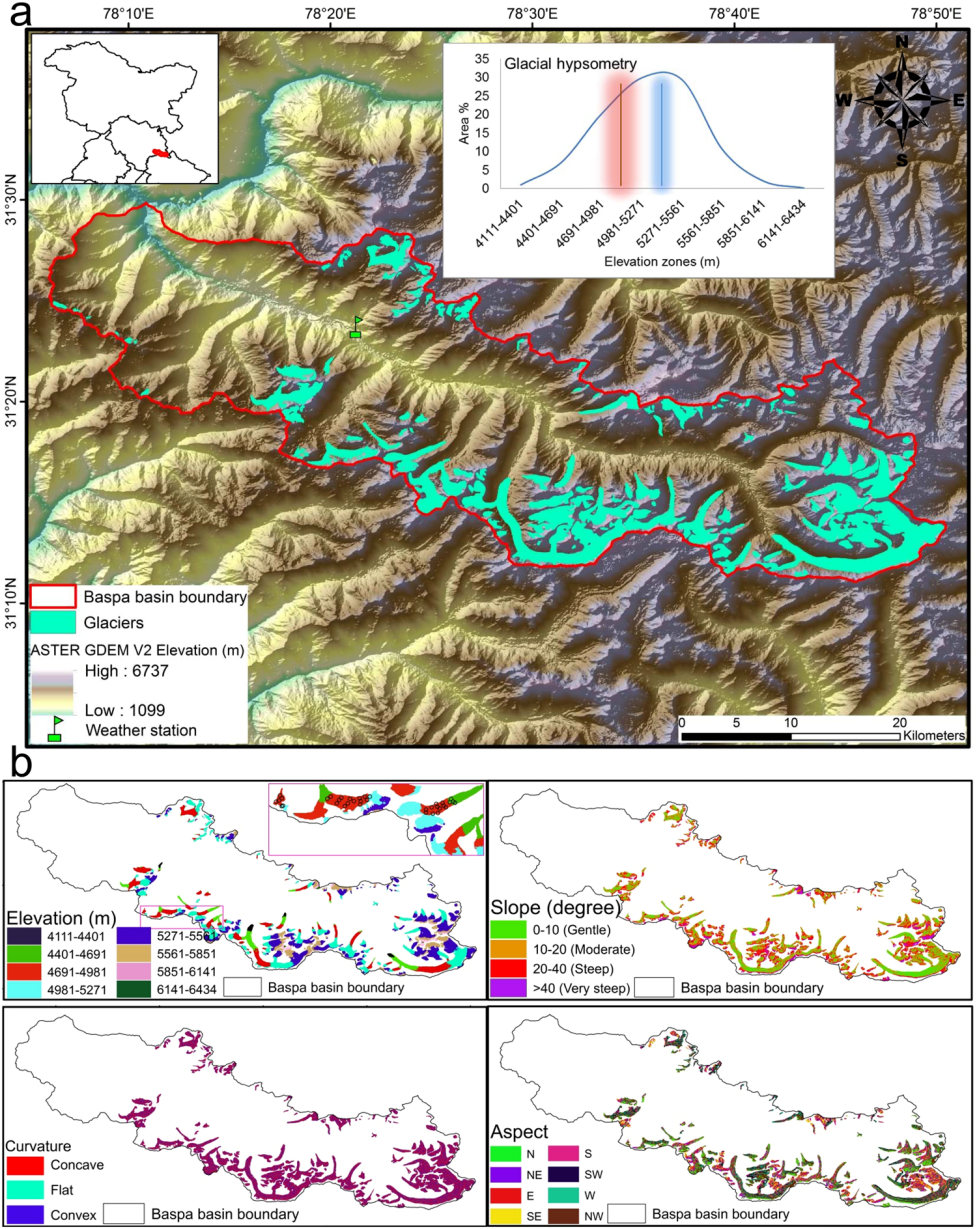
Location map and topographic conditions of the Baspa River basin. (**a**) The inset map indicates the location of the Baspa River basin (red outline) within northern India (black outline). The inset plot shows the hypsometric distribution of glaciers in the river basin. The red line and the associated shading in the inset showing the hypsometric curve depict the mean upper limit of debris cover as marked on a Landsat 8 image (Courtesy of the U.S. Geological Survey) taken on 18 September 2013 and its standard deviation (4994.41 ± 238.2 m), respectively. Similarly, the blue line and the associated shading represent the mean elevation of the transient snow line as marked on Landsat 8 images collected on 18 September 2013 and its standard deviation (5442 ± 150.7 m), respectively. The background hillshade image was generated using Advanced Spaceborne Thermal Emission and Reflection Radiometer Global Digital Elevation Model Version 2 (ASTER GDEM V2) data, which has a spatial resolution of 30 m and are the product of the Ministry of Economy, Trade, and Industry (METI) and the National Aeronautics and Space Administration (NASA). The basin boundary was derived from the ASTER GDEM V2 data using the Spatial Analyst toolbox of the ArcGIS software package, version 10.4 (http://desktop.arcgis.com/en/arcmap/10.4/get-started/main/get-started-with-arcmap.htm). (**b**) Distribution of classified topographic attributes (elevation, slope, curvature, and aspect) derived from the ASTER GDEM V2 data. The inset map within the elevation distribution panel indicates the positions of 36 stakes in total (black circles) on three glaciers within the river basin that have been monitored to estimate seasonal and yearly velocities. The Landsat image and ASTER GDEM V2 was downloaded from https://earthexplorer.usgs.gov/. All of the maps were created using the ArcGIS software package, version 10.4 (http://desktop.arcgis.com/en/arcmap/10.4/get-started/main/get-started-with-arcmap.htm).

Our study aims at determining the extent of topographic and hypsometric controls on the seasonal and annual average flow velocities of 112 glaciers of Baspa River basin in the Western Indian Himalaya (Fig. 1) that were sufficiently large to encompass at least one representative velocity pixel from the GoLIVE products and to permit the reliable extraction of second-order geomorphometric derivatives^36^ (see Methods). The glaciers in this river basin have been extensively studied for remote sensing-based mapping^37,38^, glacier dynamics^33^, glacial lakes^39^, and runoff estimations^40^. The glaciers are located within the altitudinal range of ~4,100–6,450 m and have an average mean elevation of ~5,180 m and an average mean slope of 18.2°, as derived from Advanced Spaceborne Thermal Emission and Reflection Radiometer Global Digital Elevation Model Version 2 (ASTER GDEM V2) data. We use the ASTER GDEM V2 data to derive all of the morphometric parameters used in this study because ASTER is one of the most appropriate data sources from which 3D information can be generated for cryospheric applications^36^ and the ASTER GDEM V2 data are reported to have considerable accuracy in this part of the Himalaya^41^. Furthermore, the ASTER GDEM V2 data are not affected by snow penetration issues that synthetic aperture radar (SAR)-based DEMs can display^42^. The mean upper limit of debris cover (ULDC) and the mean elevation of the transient snow line (TSL) for these glaciers, as observed towards the end of the hydrological year on 18 September 2013 Landsat 8 image for these glaciers are 4994.41 ± 238.2 m and 5442 ± 150.7 m, respectively. The total basin area is ~1,100 km^2^, ~187 km^2^ of which is glaciated; the areas of the selected glaciers vary between ~0.06 km^2^ and 33 km^2^, with a mean of ~1.67 km^2^ and a standard deviation of ~5 km^2^. Thus, there is a considerable degree of heterogeneity in the size of the glaciers in this region further making their hypsometric distributions even more relevant. The average hypsometric profile (Fig. 1a) and the derived hypsometric index (HI) of 1.13 (see Methods) suggest that most of these glaciers are either bottom-heavy or equidimensional^43^. We observe significant heterogeneity in hypso-morphometric controls on the velocities of these glaciers, the elevation being responsible for the remaining topographic parameters to differentially control the velocities. The highest glacier velocities were observed during the melting and pre-melt seasons, followed by the post-melt season, which highlights the influence of westerly-derived winter accumulation on the cumulative dynamics of the glaciers in this part of the Himalaya.

## Results

### Average seasonal and annual velocities from thresholded GoLIVE products

**Figure 2:**
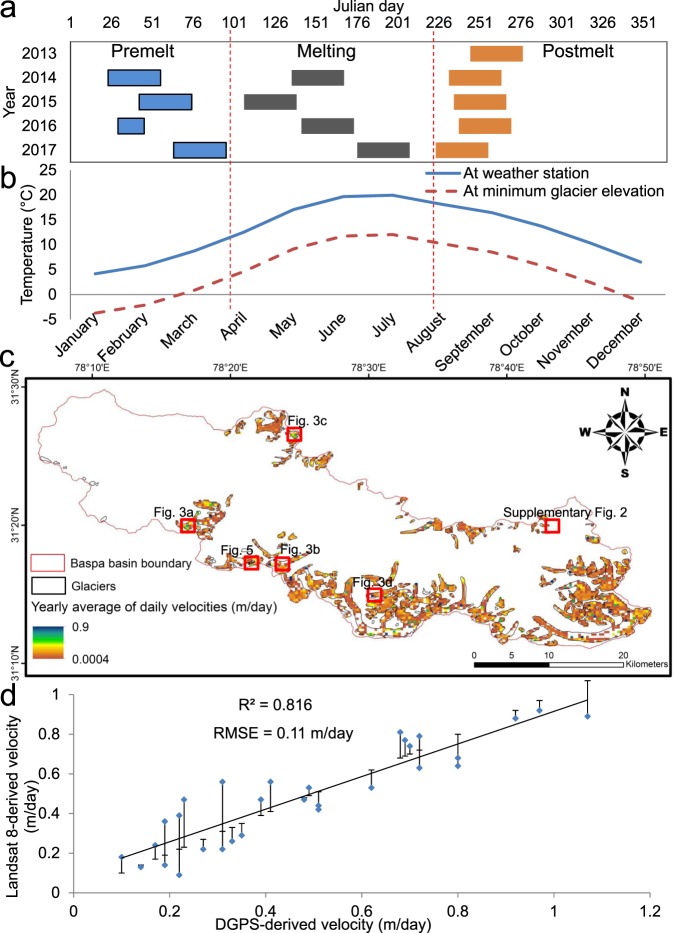
The availability of Global Land Ice Velocity Extraction from Landsat 8 (GoLIVE), Version 1 (https://nsidc.org/data/NSIDC-0710/versions/1#pan) data according to the seasons and the map of annual average of the daily velocities. (**a**) The available GoLIVE products^35^ used in this study according to the hydrological seasons of the years 2013–2017. The colour bars show the periods of time over which data are available during the different seasons. (**b**) Average of the mean monthly temperatures over 25 years (1985–2009) obtained from Rakchham weather station, which lies at an altitude of 3,045 m (Fig. 1a). A lapse rate-based^37^ extrapolation of the temperatures to the minimum glacier elevation level (4,111 m) derived from ASTER GDEM V2 is performed. ASTER GDEM V2 data is the product of the Ministry of Economy, Trade, and Industry (METI) and the National Aeronautics and Space Administration (NASA) and it was downloaded from https://earthexplorer.usgs.gov/. (**c**) Annual averages of the daily velocities for the glaciers of the Baspa River basin derived from the available GoLIVE data for 2013–2017. The blank pixels within the glacier boundaries are masked pixels with relatively low accuracies, based on the peak correlation values (corr) and the differences between the peak correlation values and the second-highest peak on the correlation surfaces (del_corr) obtained from the GoLIVE data. The red rectangles indicate the contexts of Figs. 3 and 5, and Supplementary Fig. 2. This map was created using the ArcGIS software package, version 10.4 (http://desktop.arcgis.com/en/arcmap/10.4/get-started/main/get-started-with-arcmap.htm). (**d**) A comparison of the annual average of the daily velocities from the thresholded GoLIVE datasets and *in situ* differential global positioning system (DGPS) measurements. Lengths of the error bars show the absolute errors, and the directions reflect deficits (up) or gains (down) in the Landsat 8-derived velocities relative to the field measurements.

In this study of Western Himalayan glaciers, we employed and explored a new glacier velocity product called GoLIVE (https://nsidc.org/data/NSIDC-0710/versions/1#pan)^34,35^ derived from cross-correlation of pixel positions in Landsat 8 repeat imagery. In addition to velocity component rasters, these data also contain quality control rasters, such as the peak correlation values (corr) and the differences in correlation values between the primary and secondary peaks (del_corr). Although annual average velocities are sufficient to derive key interpretations, shorter temporal repeats lead to less decorrelation between remote sensing images^44^ and allow us to extend the analyses to the seasonal scale. We performed a thorough thresholding of these data, as described in detail in the Methods section, to obtain the most reliable velocity pixels and to proceed further with the remainder of the analyses. Briefly, (i) we segregated the available GoLIVE data (Fig. 2a) according to the hydrological seasons in the study area determined from published field runoff measurements^40^ and long-term temperature records (1985–2007) from the Rakchham (3045 m asl) observatory (Fig. 2b) marked in Fig. 1a^40^. (ii) We established optimal thresholds for the corr (>0.4) and del_corr (>0.3) rasters, based on the available field velocity measurements^33,38^ for various seasons. (iii) Further, we discarded any seasonal or annual average velocity pixels that are over 2 standard deviations (2 SDs) of the velocity values, in order to use only the most reliable velocity values in our analyses. Finally, (iv) we performed Ordinary Least Squares (OLS) linear regression analyses (Supplementary Fig. 1) before and after the 2 SD thresholding to observe improvements and the degree of topographical controls as explanatory variables for velocities, and to perform additional thresholding of the pixels that show the smallest degree of topographic control by excluding ±1 SD of the standardised residuals for the OLS-modelled velocities compared to the GoLIVE velocities (see Methods). As a result of this entire preprocessing procedure, we obtain a reliable product for use in the additional analyses. Figure 2d shows the comparison between the annual averages of the daily velocities obtained from the thresholded GoLIVE datasets and the *in situ* average velocity measurements; the coefficient of determination (R^2^) is 0.82, and the root mean squared error (RMSE) is 0.11 m/day. A statistical description of the obtained yearly and seasonal averages of the daily velocities after all of the levels of thresholding have been performed is given in Table 1.

**Table 1:** The annual and seasonal averages of the daily velocities obtained after all of the levels of thresholding have been applied to the GoLIVE data.

Season	Minimum (m/day)	Maximum (m/day)	Mean (m/day)	SD (m/day)
**Averages for the entire glacier-covered area**				
Pre-melt	0.005	0.62	0.11	0.09
Melting	0.001	1.02	0.14	0.11
Post-melt	0.0004	0.58	0.06	0.06
Yearly	0.0004	0.88	0.10	0.08
**Averages for the glacier-covered area below the mean ULDC** (**4994**.**41 m**)				
Pre-melt	0.005	0.37	0.07	0.04
Melting	0.001	1.00	0.09	0.06
Post-melt	0.0004	0.22	0.03	0.03
Yearly	0.0004	0.67	0.07	0.05
**Averages for the glacier-covered area between the mean ULDC and the mean TSL** (**5442** **m**)				
Pre-melt	0.009	0.62	0.15	0.13
Melting	0.014	1.02	0.18	0.13
Post-melt	0.007	0.58	0.10	0.07
Yearly	0.011	0.88	0.14	0.12
**Averages for the glacier-covered area above the mean TSL**				
Pre-melt	0.005	0.41	0.11	0.08
Melting	0.008	0.89	0.14	0.11
Post-melt	0.0007	0.52	0.05	0.05
Yearly	0.003	0.75	0.10	0.10

**Figure 3:**
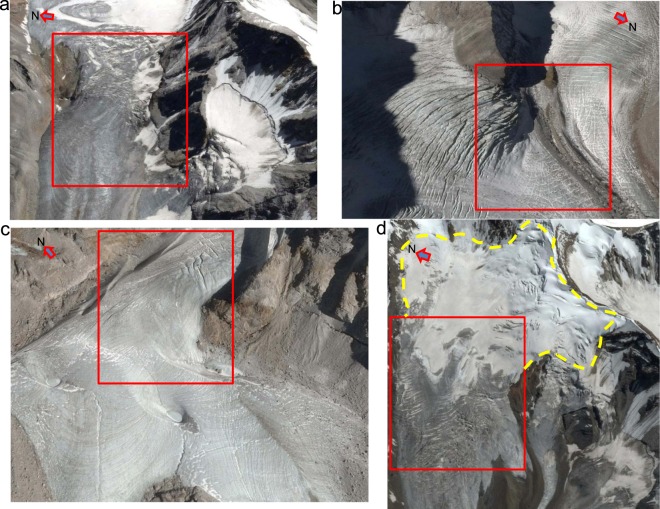
Four of the fastest-moving glaciated areas (red rectangles). All of these areas represent clean-ice zones and icefalls with heavily crevassed terrain. The contexts of (**a**–**d**) can be seen in Fig. 2c. The dotted yellow curve represents the well-developed cirque of a high-altitude (~5000–6000 m), debris-free glacier where the TSL is at an elevation of ~5390 m. Data provider for the 25 August 2014 Google Earth images used here is CNES/Airbus.

The values in Table 1 depict several characteristics of the glacier dynamics in this region of the Himalaya. First, the velocities during the melting and pre-melt seasons are significantly greater than those during the post-melt season and regulate the annual average velocities. Second, the parts of the glaciers between the ULDC and the TSL, which usually represent clean-ice zones and icefalls on most of the glaciers (Fig. 3), show the highest velocities in all seasonal and annual averages. Third, the areas above the TSL, normally representing cirque or uppermost portions of accumulation zones with presence of some snow even at the end of the hydrological year (in this case, August-September), display velocity values that are lower than those observed in the middle clean-ice parts but are higher than the debris-covered parts below the ULDC. These values and observations are largely consistent with the reported glacier velocities from this part of the world (except for the surging glaciers). Thus, these results demonstrate the high usability of the GoLIVE data, together with the proposed corr and del_corr thresholds, for monitoring the regional-scale dynamics of the Himalayan glaciers. For example, the observed average velocities in the upper and lower ablation zones of the benchmark Himalayan glacier, Chhota Shigri in the Lahul and Spiti Valley north of Baspa Valley, are >45 m/year and ~25 m/year, respectively^45^. These values agree with the results of other studies that have examined the same glacier^46,47^; the summer velocities are slightly greater than the annual averages. At high elevations in the Everest region in the Central Himalaya, average glacier velocities in excess of 40–50 m/year have also been reported, with the lower debris-covered parts displaying minimal movements^9,23,30^. For clean-ice ablation zones, the glaciers of the Everest region display velocities of less than 30 m/year^9^. Within the area between the mean ULDC and the mean TSL, which represents the upper ablation zones of the Baspa Valley glaciers, we observe mean annual velocities of ~51 m/year; the melting season velocities marginally exceed the annual average values. Similarly, for the glacier-covered area below the mean ULDC, which represents the lower ablation zone, we observe average annual velocities of ~25.55 m/year, and the flow rates show good regional congruence. This signifies that the seasonal parameters in this monsoon transition zone of the Himalaya not only complicate the glacier dynamics but they also display a significant control over glacier motion. In fact, even for the higher northern latitudes of Alaska, the seasonal velocity trends are similar; the melting season, which features relatively high values, is followed by the pre-melt and post-melt seasons^44^.

### Velocity distribution with respect to topography and the probable polythermal regime

**Figure 4:**
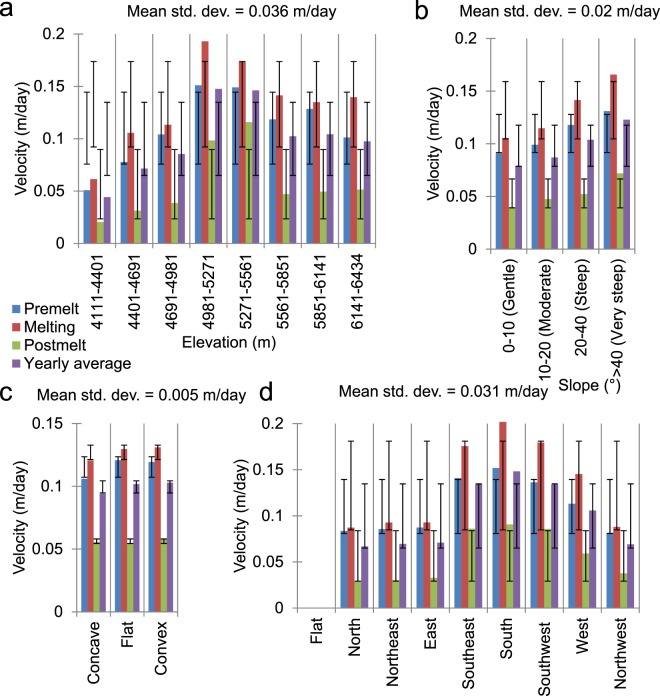
Average glacier velocities across various topographic parameters and classes derived from the ASTER GDEM V2 data. (**a**) Elevation, (**b**) slope, (**c**) curvature, and (**d**) aspect. The deviance bars show the ±1 standard deviation (std. dev.) of the velocities for different topographic classes of a given parameter within the same season and must not be misinterpreted as error bars. The mean standard deviation values are seasonal averages for a given topographic parameter.

**Figure 5:**
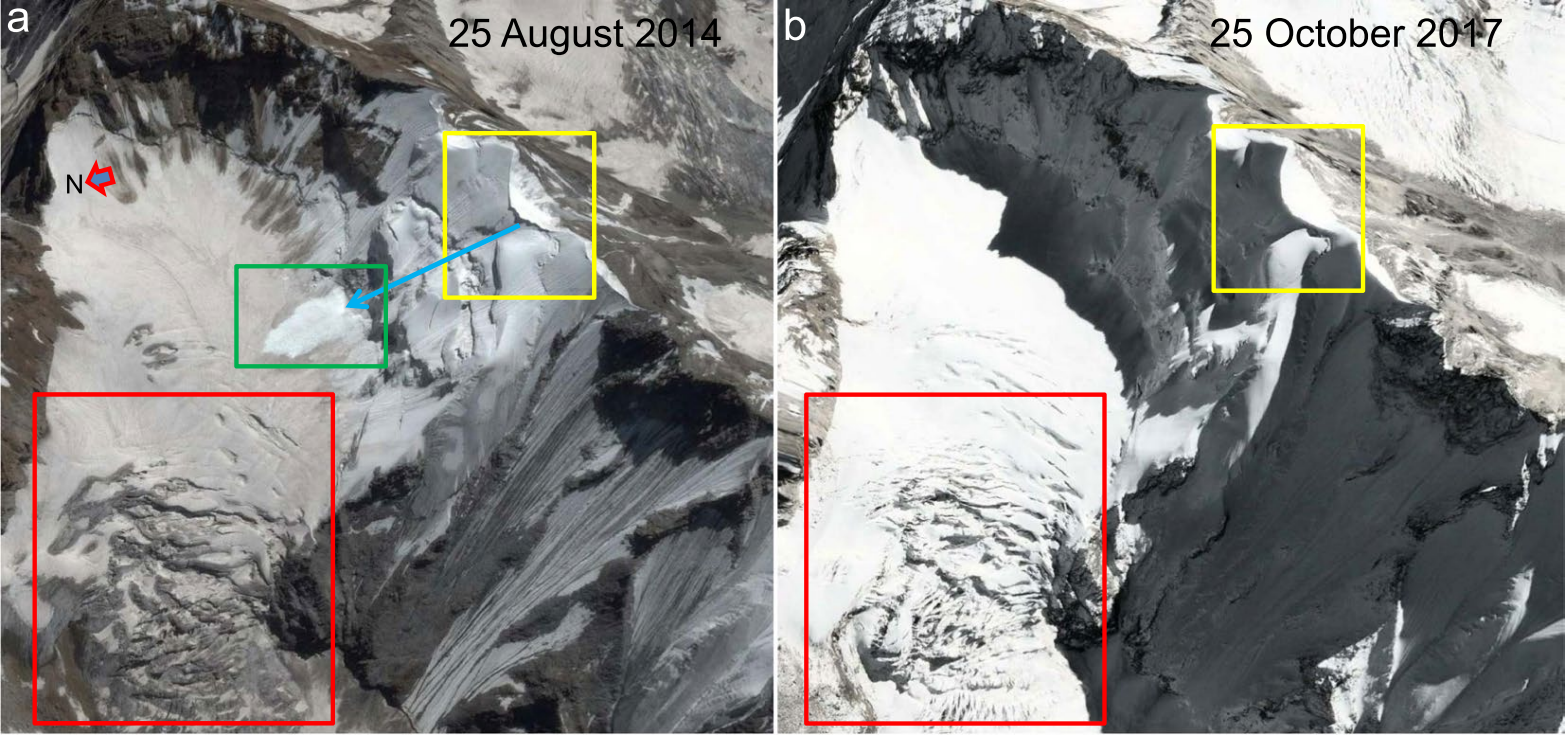
Accumulation zone of Shaune Garang glacier. (**a** and **b**) show seasonal snow-free and snow-covered periods, respectively. The contexts of (**a** and **b**) can be determined from Fig. 2c. The red rectangles show the fastest-moving part of the glacier. The yellow rectangles highlight the cornice that develops during the accumulation months and contributes significantly to the accumulation on the glacier through avalanches. The blue arrow in (**a**) shows the avalanche path, and the green rectangle indicates one such fresh avalanche event. The data provider for the Google Earth images used here is CNES/Airbus.

Further analyses of the thresholded velocity values with respect to various topographic parameters reveal the significant control exerted by elevation on glacier flow. In Fig. 4, the deviance bars show the ±1 SD ranges of the velocities for the different topographic classes of a parameter within the same season and must not be confused with error bars, even those in the following figures. The mean standard deviation values shown in Fig. 4 are seasonal averages for a given topographic parameter. The relatively high intra-season SD values and significantly greater average SD value for the elevation parameter highlights its predominant control over the glacier flow, followed by the aspect, slope, and curvature parameters. This result is understandable given that, as elevation changes, the topography and hypsometry vary and the effects of seasonal parameters, such as temperature and precipitation, become pronounced. Since this part of the Western Himalaya is situated in the monsoon transition zone^7,48^ and receives both summer monsoonal precipitation and substantial winter precipitation from the westerlies, the hypsometric profile plays a significant role in seasonal cloud accumulation and the subsequent precipitation. This significance of high-accumulation cirque zones in maintaining rapid ice flow has also been reported for several coastal glaciers in Alaska^44^. In addition, the upper reaches of Himalayan glaciers usually receive considerable contributions from avalanches^30,49,50^, which increase the ice load and the subsequent downward flow (Fig. 5). In fact, the longer glaciers in the basin can directly be correlated to their higher accumulation zones, which are vital for sustaining persistently flowing ice streams in the lower reaches of these glaciers. A similar type of climate regime has also been observed in the Mount Everest region^48^, and the degree of elevation control on glacier flow is reported to be immense^30^, as the glaciers fed by high-altitude cirques possess substantial amounts of active ice. The reported velocities for the longest Himalayan glacier, Siachen glacier in the Karakoram also display strong elevation control^51^ as, in this part of the Himalaya, westerlies contribute significantly to accumulation, and several surging glaciers have been reported to result from combined effects of the local climate and topography^52^.

The elevation classes shown in Fig. 4a were not selected randomly; instead, they are based on three main factors. First, in order to avoid sampling bias, we established classes with equal intervals and optimal sizes so that they would all encompass a sufficient number of reliable thresholded velocity pixels, which is extremely crucial for the hypso-morphometric analyses presented in the next section. Second, with 8 classes of equal intervals, we obtained an elevation rise of ~290 m per class, which also provided an approximate temperature change of ~2 °C within each class, based on the published seasonal lapse rate for the study area^37^. This separation was vital in differentiating the various thermal regimes over the glaciers and their control on the flow rates. Third, and most importantly, the ~290 m per class range accounts for the ±150.7 m SD of the TSL and provides a definite class (5271–5561 m) above it, where the majority of the glaciers in the basin have their accumulation zones. Similarly, it also establishes that the classes 4981–5271 m (ULDC) and 5271–5561 m represent clean glacier ice or icefall zones for the majority of the glaciers in the basin (Fig. 3). In Fig. 4a, it is apparent that the observed seasonal and annual averages of the daily velocities are considerably higher for these middle elevation zones. In addition, for all of the elevation zones, we clearly observe a constant seasonal pattern in the velocities (melting > pre-melt > post-melt). For the post-melt season, apart from the clean-ice zones, the velocities do not show significant variations in either the debris-covered parts or accumulation zones, where the velocities vary between ~0.02 and 0.05 m/day; on the other hand, for the velocities in pre-melt and melting seasons in these zones, the deviation ranges from ~0.05 to 0.14 m/day. This result shows the importance of both winter snow loading followed by rapid surface melting, in regulating glacier velocities in this part of the Himalaya. These results further indicate that the sliding motion may not be the only important factor; instead, deformational flow generated by the accumulated winter snow loading provided by the westerlies is equally vital in controlling glacier movements in the Western Himalaya. During the pre-melt seasons, episodes of the westerlies are quite frequent in these mountains; when the weight of this newly deposited snow exceeds the carrying capacity of the weakest layer of the snow on a slope, it causes significant creep, as well as snowpack fractures and avalanches^53^.

The exact thermal regimes of these glaciers were previously unknown until now, owing to the research and data deficiencies. Due to the results of the present study, however, we can get a clue about these thermal regimes. As mentioned above, the average annual velocities and their patterns in these mountains match closely with those of the glaciers in the Mount Everest region^9,23,30^, which have been reported to display polythermal regimes^54–58^. Based on Weertman’s sliding law^59^, which describes both pressure melting and creep-rate enhancement driven by stress concentration as factors in producing noticeable glacier motion, we can safely derive a preliminary analogy regarding the polythermal regimes of a majority of the Western Himalayan glaciers.

Active fractures, such as crevasses and moulins, are known to be prevalent on such polythermal glaciers carrying supraglacial meltwater into the polythermal englacial environment^60^ and facilitating the typical polythermal glacier flow patterns as described above. The glaciers in the Western Himalaya have been reported to possess significant proportions of crevasses, owing to the differential glacial motion (Figs 3 and 5), and seasonal dynamics related to their orientations and creep^38^. One of these seasonal dynamics is the cyclicity in crevasse orientations, which has been reported from a glacier in the Baspa River basin^38^, thus highlighting the significant roles of crevasses in maintaining polythermal glacier flow regimes. Such dynamics of refreezing or annealing of surface crevasses and fractures have previously been attributed to the polythermal controls on the glacier velocities in the Arctic, as well as in the Himalaya^61–66^. Figure 4b shows a gradual increase in the velocity with increasing surface slope, which favours both sliding motion and deformational flow produced by the accumulated winter snow loading. Similarly, the south-facing (S, SE, and SW-facing) slopes on the glaciers show significantly higher velocities than the other slopes (Fig. 4d), probably because these slopes are most prone to year-round melting in the Himalayan context. Nevertheless, the fact that the velocities on these south slopes are closely followed by the velocities on the west slopes also emphasises the importance of snow accumulation in driving glacier flow. The south-facing slopes are more susceptible to receiving the maximum amounts of snow during the monsoon season and are also vulnerable to the subsequent melting, as they receive direct sunlight over the maximum duration on any given day in the same season. Similarly, the west-facing slopes are more likely to receive contributions from the westerlies in the winter and drive the deformational flow in the pre-melt and melting seasons. To understand Fig. 4c, an understanding of the flat curvature is important; it should not be confused with a flat slope or the aspect. A flat curvature does not primarily reflect a flat slope; instead, it includes even slopes with significant inclinations and represents the shape or curvature of the slope. The surface curvature parameter in Fig. 4c further strengthens our premise that glacier motion in this part of the Himalaya is driven equally by surface melting and surface loading. The post-melting season, when the snow loading is minimal, shows nearly similar velocity values for all three curvature classes (Fig. 4c). However, velocities with nearly double these values are observed during the other two seasons, which are more favourable for snow loading and melting. Furthermore, the fact that the average velocities displayed by the flat and convex surface classes, which are more susceptible to instabilities and deformational motion due to snow loading^50,53^, are greater than the velocities observed for the concave curvatures illustrates the significant controlling forces exerted by snow seasonality.

### Hypso-morphometric variations in velocity distribution

**Figure 6:**
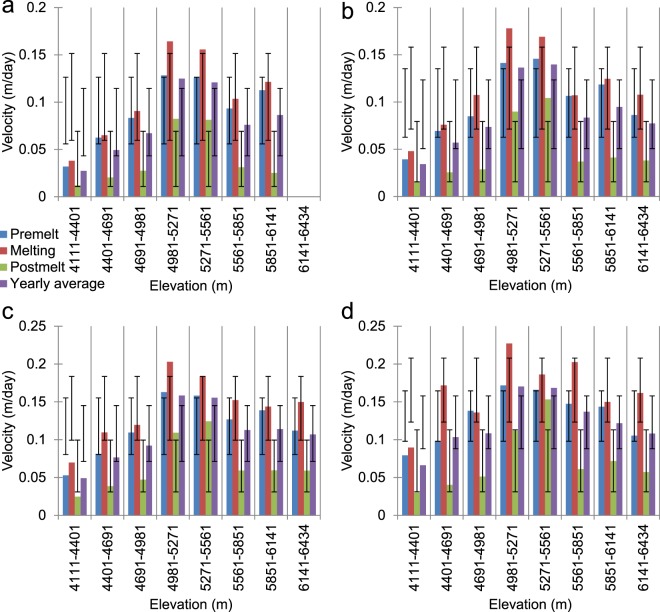
Average glacier velocities across various slope classes within different elevation zones derived from the ASTER GDEM V2 data. (**a**) 0°–10° (gentle), (**b**) 10°–20° (moderate), (**c**) 20°–40° (steep), and (**d**) >40° (very steep). The dispersion bars show the standard deviation ranges of the velocities for a given slope class with different elevations within a season.

**Figure 7:**
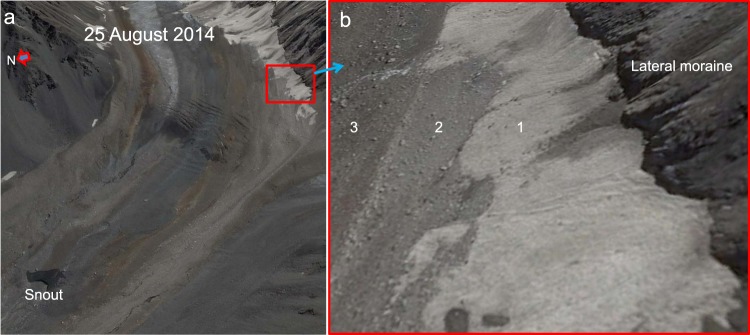
Steep, thin, debris-covered, lower-elevation regions (red rectangle) adjacent to the terminal moraine of Shaune Garang glacier, as seen in a Google Earth image collected on 25 August 2014. (**a**) Shows an overview, whereas (**b**) displays a zoomed-in view of the thin debris regions. Zone 1 in (**b**) represents dirty but exposed ice, zone 2 represents a thin layer of debris on the steepest slopes, and zone 3 represents the increase in debris thickness as the slopes become gentler. The data provider for the Google Earth image is CNES/Airbus.

Based on the results discussed in the previous section, the greater impact of elevation over other morphometric parameters becomes evident, for the intuitive reason that elevation changes also account for temperature and precipitation variations and facilitate the separate consideration of debris-covered, clean-ice, and snow-covered zones. Although the plots in Fig. 4 yield some vital clues regarding the effects of topography, re-examining them using a hypso-morphometric approach that considers slope, aspect, and curvature classes separately for different elevation zones (Fig. 4a) helps us to further untangle the complexities of the effects of topographic variables. For example, Fig. 6 represents one such hypso-morphometric distribution of the velocities across various slope classes in different elevation zones. The pattern of the velocities across different elevation zones is similar for all of the slope classes; however, for steeper slopes (Fig. 6c,d) the magnitudes of the velocities clearly become pronounced at middle elevations that contain clean-ice zones. The 0°–10° (gentle) slopes are not present at the highest elevation zones, which mainly represent the steep headwalls that are prone to avalanching. The velocities that correspond to the post-melt season shown in Fig. 4b do not show significant deviations. Even in Fig. 6, the noticeable deviations in the velocities seen in the post-melt season are observable only in the middle elevation classes unlike the velocities in rest of the seasons where velocity changes are noticeable in all of the elevation classes. One anomalous observation can be made for the melting season velocities at relatively low elevations (4401–4691 m) on the steepest slopes (Fig. 6d). The exact reason for this increase in the velocities in the debris-covered parts during the melting season is difficult to determine. However, it may be related to the fact that thick debris cover considerably impedes melting^40^; the areas with the steepest slopes between 4401–4981 m typically represent areas at lower elevations that adjoin lateral moraines and have comparatively thin or no debris to hamper melting or flow during the snow-free months (for an example, see Fig. 7). Interestingly, such regions with limited debris are absent in the lowest elevation zones that contain the heavily debris-covered snouts of nearly all of the glaciers (for an example, see Fig. 7).

**Figure 8:**
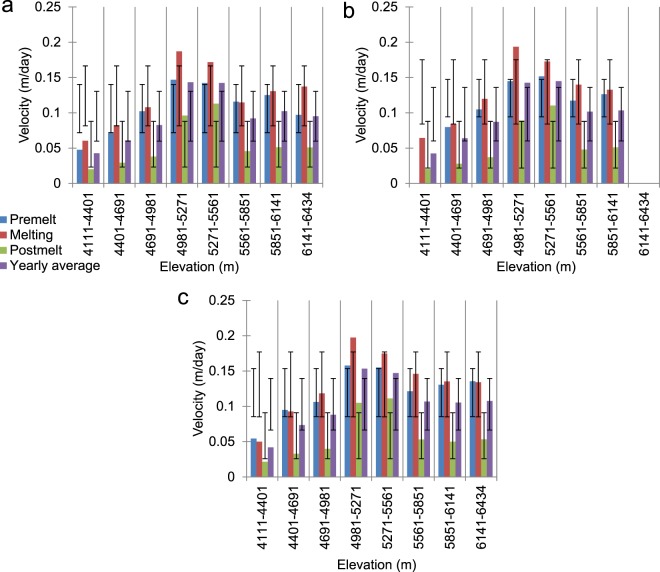
Average glacier velocities across various curvature classes within the different elevation zones derived from the ASTER GDEM V2 data. (**a**) Concave, (**b**) flat, and (**c**) convex. The dispersion bars show the standard deviation ranges of the velocities for a given curvature class at different elevations within a season.

**Figure 9:**
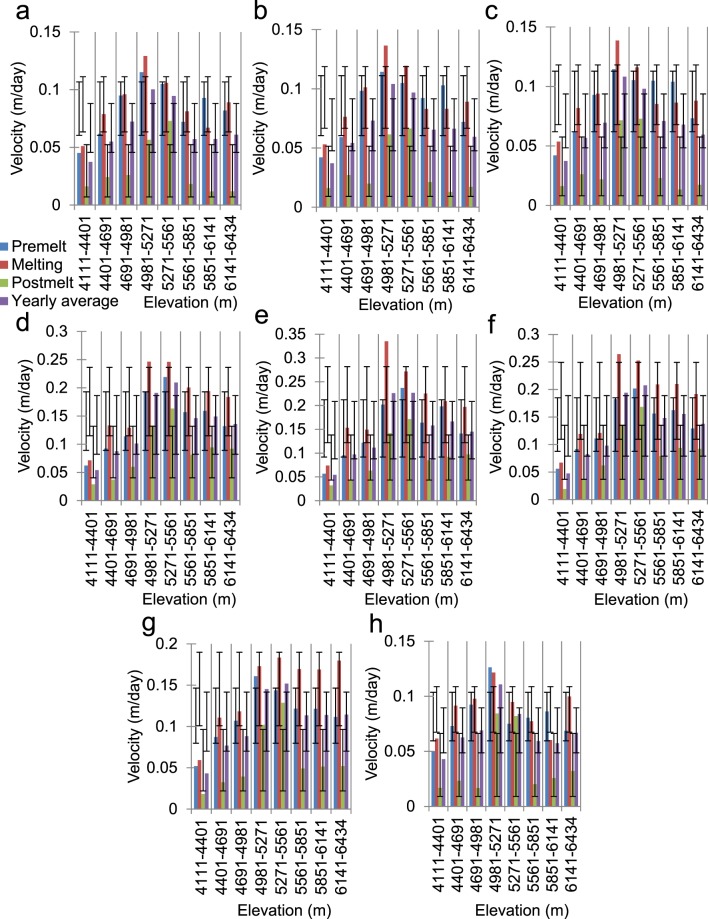
Average glacier velocities across various aspect classes within the different elevation zones derived from the ASTER GDEM V2 data. (**a**) North, (**b**) northeast, (**c**) east, (**d**) southeast, (**e**) south, (**f**) southwest, (**g**) west, and (**h**) northwest. The dispersion bars show the standard deviation ranges of the velocities for a given aspect class at different elevations within a season.

Figure 8 displays similar hypso-morphometric velocity distributions for the three curvature classes. The flat curvatures cannot be observed for the highest elevations, whereas valid velocity pixels are absent for the flat curvatures seen at the lowest elevations in the pre-melt season (Fig. 8b). The general patterns of the velocities across the elevation ranges are as expected (middle elevations > higher elevations > lower elevations). However, one relevant conclusion can be drawn from this distribution regarding the pre-melt season velocities for convex curvatures at the lowermost and uppermost elevations (Fig. 8c), where the presence of thick debris or the lowest temperatures, respectively, discourage melting most strongly. The pre-melt velocities are the highest at these elevations, due to the significant controlling effect of unstable convex curvatures^50,53^ in the snow-covered months, which overcomes the non-melting factors and likely promotes deformational motion during the winter months. Taking a closer look at the aspect profile shown in Fig. 9, several inferences can be obtained that are not directly evident from Fig. 4d. Specifically, in general, the pattern of the velocities is as expected, i.e., middle elevations > higher elevations > lower elevations, and melting > pre-melt > post-melt. However, several seasonal and altitudinal patterns of variation are worth discussing. For example, we see considerable numbers of instances in which the pre-melt velocities exceed the melting season velocities at higher elevations for the aspects that do not directly face the sun (Fig. 9a–c,h) and are not prone to high melting. These results hint at the dominance of deformational flow at the local scale. Similarly, the sun-facing aspects (Fig. 9d, e, f) display the highest velocities of all of the elevation zones and seasons, thus highlighting the significant influence of glacier melt on ice flow. Moreover, the magnitude of the deviations from the seasonal means is significantly higher for these sun-facing aspect classes (these values even exceed 0.1 m/day in most instances) across the lower and middle-to-high elevation zones. This result further emphasises that, in the absence of debris cover, melting-induced glacier flow can increase exponentially; in several cases, it even triples (Fig. 9d–f).

## Discussion

The outcomes of this study are relevant in several respects regarding our understanding of the complex dynamics of Himalayan glaciers at the regional scale. In addition to the regular glacier facies^67,68^, the glaciers in this part of the Himalaya are significantly debris-covered^37^. As we observe in the present study, although the geomorphometric parameters play important roles in regulating the glacier flow, their contributions are significantly enhanced or subdued during various seasons and under different melting conditions within the different glacier facies or debris-covered regions. The significance of debris cover in driving glacier dynamics in the Himalaya is poorly explored and has been neglected in modelling future runoff and sea-level changes^32^, primarily due to a lack of field data. However, for a start, it is important to quantify and qualitatively observe the effects of debris cover on glacier flow using reliable satellite-based data, and the present study indicates that the GoLIVE data appear to be an easily available and useful product for performing glaciological research at large spatial and temporal scales. A crucial contribution of this work involves identifying the proper cross-correlation thresholds to refine the GoLIVE data for Western Himalayan glaciers. After applying all of the levels of refinements, none of the studied glaciers show any anomalous pattern or magnitude of flow that should be discussed in detail. Therefore, this cumulative basin-scale analysis of the glaciated areas provides meaningful and sequential information about the primary controls for glacier velocities in the Western Himalaya. These methods to analyse seasonal velocity patterns for the Western Himalayan glaciers are demonstrated here as a short-term analysis (2013–2017) based on the GoLIVE data availability and can further be implemented on longer-time scales to (i) predict glacial changes and to issue disaster warnings, and (ii) to assess the role of other time-varying factors (including climate change, albedo changes, crust deformations, anthropogenic activities, and earthquakes) in controlling the glacier dynamics.

Another conclusion of this work is that, through an analogy with the published velocity records and patterns from the Mount Everest region and the Bhutan Himalaya, we are able to draw the preliminary conclusion that the flow of most Western Himalayan glaciers in the monsoon transition zone might be governed equally by both melt-facilitated sliding and deformational creep; moreover, these glaciers display the characteristics of polythermal glaciers. However, we are aware that exceptions may exist at local scales and for low-altitude glaciers and bottom-heavy glaciers with considerable debris cover. The lower-elevation margins and surface ice of polythermal glaciers are typically cold-based, whereas the higher-elevation accumulation regions are warm-based^69^; thus, glacier movement takes place via both basal sliding and subglacial deformation in the accumulation area of polythermal glaciers, but it is driven almost entirely by deformation within the cold-based, lower-elevation portions of polythermal glaciers. However, the presence of excess debris cover (as is seen in the Western Himalaya) can further increase the complexity of glacier flow in polythermal regimes. These factors and the developed stresses can together lead to the deformation of proglacial permafrost, forming ice-cored moraines and rock glaciers (Supplementary Fig. 2)^69–71^. As highlighted in Supplementary Fig. 2, such proglacial permafrost landforms are abundant in the study area and provide further evidence of the possible polythermal nature of these glaciers.

In this study, we have also proposed a hypso-morphometric approach that can be used to decipher the effects of topography in greater detail, and the usefulness of this technique has already been discussed in the results section. However, one additional benefit of this approach is its minimal dependence on the type of DEM selected to derive the topographic parameters, as the entire hypso-morphometric analysis is performed after dividing the parameters into several classes, which is essentially an averaging approach. To assess the biases that may enter into these analyses due to the use of a particular type of DEM (here the ASTER GDEM V2 data), as a control measure, we attempted to perform a similar analysis using a newly released DEM that is constitutionally different from the ASTER GDEM V2 data and is reportedly the most precise global-scale elevation data^72,73^. This dataset is named the Advanced Land Observing Satellite (ALOS) Global Digital Surface Model (ALOS World 3D - 30 m; AW3D30). Although at very local scales, we observed some topographic differences between the two DEMs (for example, mean elevation differences of up to ~9 m), for the averaged velocity versus hypso-morphometric plots, the velocity patterns match those derived using ASTER GDEM V2 closely; thus, we do not include those plots in the results presented here. Moreover, a comparison of both of the DEMs was not an objective of the present research and could easily amount to a separate research topic.

The debris-covered Himalayan glaciers, which receive a significant amount of winter accumulation through the westerlies, are less sensitive to minor temperature increases and melting^56^; indeed, the results presented in this study show the combined effects of the seasons and topographic parameters on the glaciers. However, for the debris-free parts of the glaciers, topography plays a vital role in determining the surface energy balance, which ultimately affects the melt rate^74^. Therefore, when performing glacio-hydrological modelling over long time periods, it is not ideal to consider the topography to be temporally constant. In fact, an inverted ablation gradient in the lower glacier reaches has been reported for the polythermal glaciers in the Everest region that is due to increases in debris thickness, thus promoting mid-glacier mass loss^75^. This effect is causing localised surface lowering at the middle elevations; it is ultimately changing the topography and reducing the glacier velocities, further contributing to the stagnation of the glacier tongues^75^ and making them prone to detachment. Based on the extent of the effects of the topographic variables that we observe in the present study, we strongly recommend incorporating dynamic topography that reflects the constant evolution of glaciers in models to generate more precise forecasts. The advent of UAVs in glaciology^25^ provides such opportunities of generating repeated low-cost, high-resolution topographies with unprecedented accuracies and can contribute extensively to the Himalayan glaciological research. Changes in glacier dynamics can significantly affect the dynamics of suspended sediment load with respect to seasonal discharges and temperatures in the Himalayan glacierised catchments^76^ and since the lower reaches of Baspa River basin are home to several proposed and running hydroelectric projects^76^, understanding glacier dynamics with respect to different seasons and its effect on the sediment load becomes extremely relevant as one of the future scopes of our study.

## Materials and Methods

### Glacier boundaries

We used the glacier outlines of the glaciers in South Asia West (Region 14) of Version 5.0 (V5) of the Randolph Glacier Inventory (RGI)^77^ (https://www.glims.org/RGI/rgi50_dl.html) as the base data. We reprojected the RGI V5 glacier outlines from the geographic coordinate system (GCS) using the World Geodetic System (WGS) 1984 datum to WGS 1984 Universal Transverse Mercator (UTM) coordinates, zone 44N, to ensure their compatibility with the GoLIVE products used here. These glacier outlines are based on satellite images collected in August and September of 2000 and 2001. Because the GoLIVE data used in our study extend from 2013–2017, we updated the outlines using a Landsat 8 image collected on 18 September 2013 (id: LC81460382013261 LGN00) and performed additional refinements using recent high-resolution Google Earth images taken during the snow-free months that cover the study area and field validation, following the well-defined Global Land Ice Measurements from Space (GLIMS) guidelines^78^. Finally, we decided to include in the analyses only those glaciers with total areas of >0.045 km^2^ (a total of 112 in the present case), due to the limited spatial resolution of the GoLIVE data. This threshold also helped us to eliminate very small glaciers or glacierets, as second-order geomorphometric derivatives might not be very reliable for very small glaciers^36^.

### Geomorphometric parameters

To derive the geomorphometric parameters, we employed the *Slope*, *Aspect*, and *Curvature* tools within the *Spatial Analyst* toolbox of the ArcGIS software package, version 10.4 (http://desktop.arcgis.com/en/arcmap/10.4/get-started/main/get-started-with-arcmap.htm). For a given grid cell, the *Slope* tool calculates the maximum rate of change in value from that grid cell to its eight neighbouring grid cells, whereas the *Aspect* tool estimates the direction of the slope in terms of the maximum rate of change in value from each grid cell to its eight neighbours^79^. The *Curvature* tool computes the second-order derivative of the input surface on a per-grid-cell basis for each grid cell, for which a fourth-order polynomial is fitted to a surface composed of a 3 × 3 window^80,81^. The reclassification of these parameters was performed using the Reclassify tool within the *Spatial Analyst* toolbox of ArcGIS 10.4.

### GoLIVE data selection and filtering

A multi-level filtering of the GoLIVE data was performed to ensure that we would proceed through the analyses described in this paper using only the pixels that yielded the most accurate/reliable velocity estimates. For details on the GoLIVE data and suggestions from the developers on its proper utilisation, interested readers can refer to the detailed data description given within User Guide at https://nsidc.org/data/NSIDC-0710/versions/1#pan. These data are already well-filtered and processed (using high-pass spatial filtering, normalised cross-correlation, resampling, correlation strength analysis, sub-pixel offset determination, and adjustment of geolocation errors), and these procedures substantially suppress the errors that arise due to changing snow, cloud cover, and lighting conditions for the pair of images used. In addition, we performed several selection procedures to select the most valid pixels for our research objectives.

First, we decided to use datasets with a relatively short temporal span for the present analyses. For short temporal spans of 16–32 days, the reported accuracy even without additional thresholding can be as high as ~0.02 m/day, as short temporal repeats lead to less decorrelation between remote sensing images^44^. Therefore, we used data with temporal spans of 16–32 days (Fig. 2a), which also helped us to carry out precise analyses at the seasonal scale. The GoLIVE data are arranged according to Julian day, and the Julian days utilised by us in different years are marked in Fig. 2a.

Second, we determined optimal thresholds for the corr and del_corr rasters, which are provided with the GoLIVE datasets. The corr and del_corr rasters describe the confidence and accuracy of the vector displacement, respectively, and the suggested thresholds given in the GoLIVE User Guide for high confidence and high accuracy are corr >0.3 and del_cor >0.15. However, the developers provide the corr and del_corr field rasters to permit users to investigate the optimal threshold values appropriate for their study area. We first started with different permutations and combinations of the corr and del_corr value thresholds, matched the results with published field velocity measurements derived from DGPS observations^33,38^ for various seasons, and finally selected the thresholds corr >0.4 and del_corr >0.3. These thresholds are optimal in the sense that they provide reliable velocity estimates (Fig. 2d) and minimise the loss of velocity pixels after thresholding; thus, extensive data gaps or void areas are not produced (Fig. 2c). To implement these thresholds for obtaining the final velocity rasters, we used the *con* expression within the *Spatial Analyst Raster Calculator* tool in ArcGIS, version 10.4.

Third, we discarded any seasonal or annual average velocity pixels that exceeded 2 standard deviations (2 SD) of the velocity values in order to use only the most reliable velocity values in our analyses. Further, we performed an OLS linear regression analysis to assess the level of dependency of the velocity values on the topographic parameters. The purpose of this OLS analysis should not be mistaken as our prime objective or as a step that is crucial in obtaining the results presented in this study. Instead, this analysis (which yielded an adjusted R^2^ value of ~57%) was a first step, and the results hinted that the velocities of these glaciers are not exclusively driven by topography; instead, they are driven by a complex and heterogeneous mixture of debris cover, the seasons, elevations, and the geomorphometric parameters. The OLS linear regression analysis (Supplementary Fig. 1) was performed twice, before and after the >2 SD thresholding. The second round of OLS linear regression was performed to assess the improvements associated with the thresholding and the degree to which the topographic parameters explained the velocities, and to perform an additional thresholding of the pixels that showed the least topographic control by excluding ±1 SD of standardised residuals for OLS-modelled velocities as compared with the GoLIVE velocities. The OLS linear regression also helped us to observe the spatial distribution of the standardised residuals; we observed that they were evenly distributed across the river basin in all of the terrain parameters and classes (Supplementary Fig. 1c) and showed no spatial clustering that would have led to significant biases. The OLS tool is embedded in the *Spatial Statistics* toolbox of version 10.4 of the ArcGIS software package and requires dependent (velocity in the present case) and explanatory (elevation, slope, aspect, and curvature in the present case) variables in the form of an attribute table for a single feature class. To generate the feature class with all of the needed variables, we followed the approach suggested by Bhardwaj *et al*.^82^. We used the *Raster to Point* tool in the *Conversion* toolbox of version 10.4 of the ArcGIS software package to generate point shape files for the velocity rasters. Further, we used the *Extract Values to Points* tool in the *Spatial Analyst* toolbox of version 10.4 of the ArcGIS software package to extract the corresponding explanatory variables for each of the points. This feature class was then provided as input to the OLS tool, and the processing was carried out without any error message regarding spatial clustering, which can affect model performance. The histograms of the OLS-derived standardised residuals (model over- and under-predictions) of the predicted velocities before and after removing the >2 SD satellite-derived velocity values that are shown in Supplementary Fig. 1a and the improvements in the plots of the standardised residuals in relation to the OLS-predicted velocities before and after the >2 SD thresholding that are shown in Supplementary Fig. 1b highlight the validity of our approach in removing these outliers. Supplementary Fig. 1c shows the spatial distribution of the standardised residual values; blue dots represent under-predictions and red dots represent over-predictions beyond the 1-SD limits, thus highlighting the areas where the velocities could not be modelled accurately using the OLS approach. Thus, we preferred to discard such pixels because we wanted to specifically study the extent to which topographic variables affect velocities. To meet that goal, we needed the velocity pixels that display a dependence on the topographic parameters within a certain level of confidence.

Fourth, we finally averaged out the seasonal velocity values of the thresholded rasters for all of the years to proceed with the analyses. Furthermore, as the last step in the preparation of the GoLIVE database, we used the *Zonal Statistics as Table* tool within the *Spatial Analyst* toolbox of version 10.4 of the ArcGIS software package to summarise the values of the final velocity rasters within the reclassified zones of the morphometric rasters. The results were output to tables to permit the generation of the statistical plots and hypso-morphometric analyses presented in this paper.

### Hypsometric Index (HI)

To perform the HI calculations, we used the formula suggested by Jiskoot *et al*.^43^ that is given below as equation 1.

1 $${HI}=\frac{{Maximum}\,{elevation}-{Mean}\,{elevation}}{{Mean}\,{elevation}-{Minimum}\,{elevation}};{\rm{if}}\,0 < {\rm{HI}} < 1,\,{\rm{then}}\,{HI}=\frac{-\,1}{{HI}}$$

Based on the thresholds suggested by Jiskoot *et al*.^43^, five categories of glaciers exist: (1) very top-heavy (HI < −1.5), (2) top-heavy (−1.5 < HI < −1.2), (3) equidimensional (−1.2 < HI < 1.2), (4) bottom-heavy (1.2 < HI < 1.5), and (5) very bottom-heavy (HI > 1.5). For our case, the combined HI was 1.13, which is at the boundary between equidimensional and bottom-heavy profiles (i.e., the glaciers in this river basin are predominantly bottom-heavy or equidimensional).

### Ethical approval and informed consent

We confirm that this study is based entirely on remote sensing methods and does not involve any biological experiments.

## Electronic supplementary material

Supplementary information

## Data Availability

All of the datasets used in the analyses presented here are freely available, and the references have been provided at appropriate places within the paper.
