# Supplementary material for: Heterogeneity in topographic control on velocities of Western Himalayan glaciers
Source: Sci Rep. 2018 Aug 27;8:12843. doi: 10.1038/s41598-018-31310-y (PMC6110744; doi:10.1038/s41598-018-31310-y)
Supplement: Supplementary file 1 — Supplementary information [file 41598_2018_31310_MOESM1_ESM.pdf]

Supplementary information

Heterogeneity in topographic control on velocities of Western Himalayan glaciers

Lydia Sam[1,2,3]*, Anshuman Bhardwaj[3], Rajesh Kumar[2], Manfred F. Buchroithner[1], F. Javier Martín-Torres[3,4,5]

[1]Institut für Kartographie, Technische Universität Dresden, Germany
[2]Department of Environmental Science, Sharda University, Greater Noida, India
[3]Division of Space Technology, Department of Computer Science, Electrical and Space Engineering, Luleå University of Technology, Luleå, Sweden
[4]Instituto Andaluz de Ciencias de la Tierra (CSIC-UGR), Armilla, Granada, Spain
[5]UK Centre for Astrobiology, School of Physics and Astronomy, University of Edinburgh, UK

*Corresponding author: Lydia Sam (lydiacsam@gmail.com)

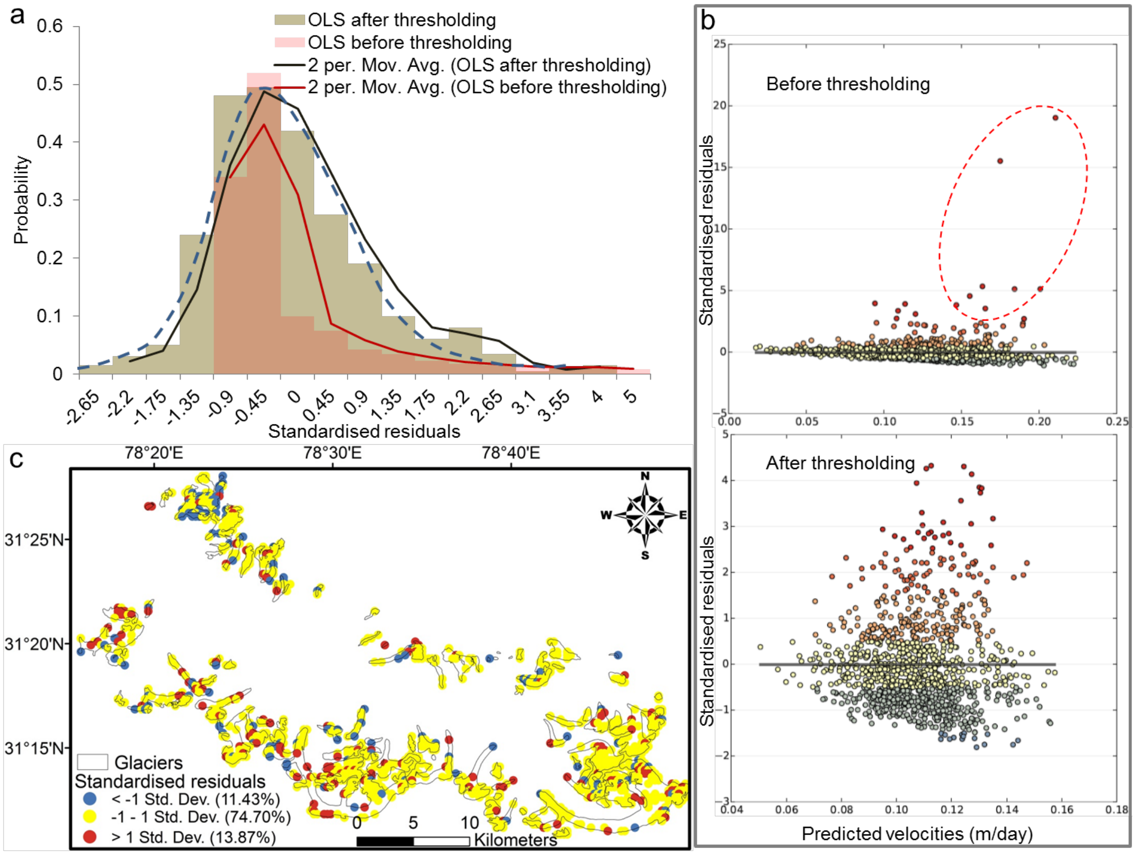

Supplementary Fig. 1 | Results of ordinary least squares (OLS) linear regression with an adjusted R^2 of ~57%. (**a**) Histograms of the OLS-derived standardized residuals (model over- and under-predictions) of the predicted velocities before and after removing the satellite-derived velocity values that exceed 2 standard deviations. The moving average curves represent the skewness with respect to a normal distribution (dashed blue curve). (**b**) Plots of standardized residuals in relation to the OLS-predicted velocities before and after removing the satellite-derived velocity values that exceed 2 standard deviations (which correspond to the residual values within the dotted red ellipse). (**c**) Spatial distribution of the standardized residual values. Blue dots indicate under-predictions, whereas red dots represent over-predictions beyond the 1 standard deviation limits.

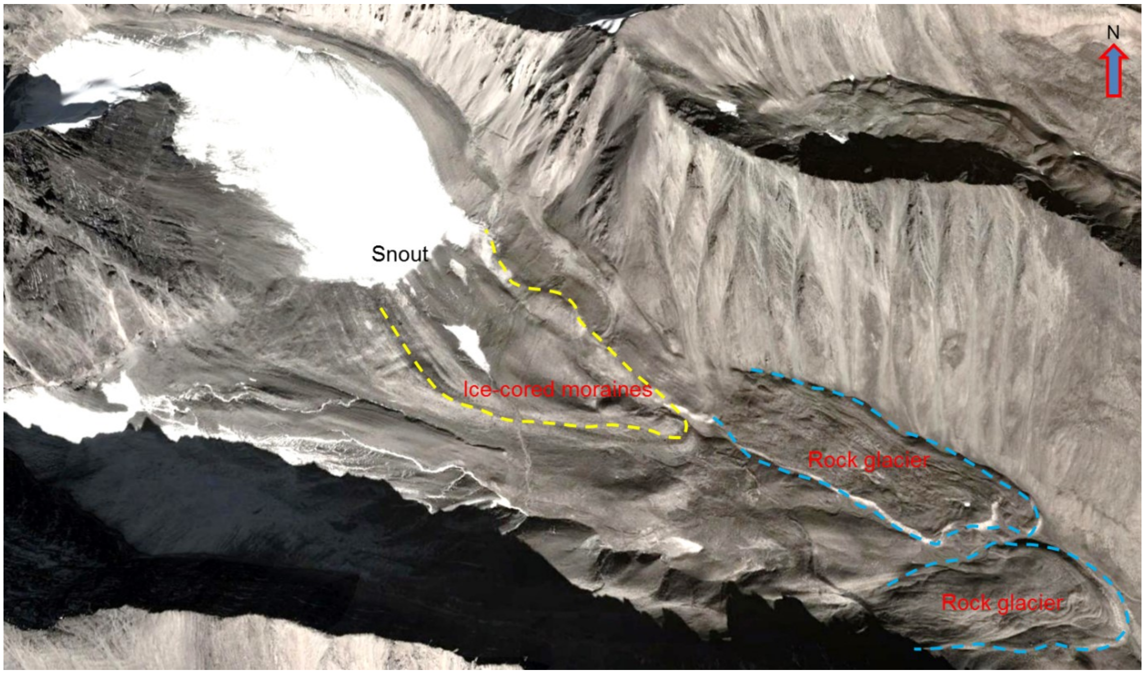

Supplementary Fig. 2 | Proglacial permafrost occurring as ice-cored moraines (dashed yellow curve) and rock glaciers (dashed blue curves). The contextual information for this glacier can be determined from Fig. 2. This Google Earth image was collected on 17 December 2016, and the data provider is CNES/Airbus.
